# Nanopore Genome Sequencing and Variant Analysis of the Susceptible *Candida auris* Strain L1537/2020, Salvador, Brazil

**DOI:** 10.1007/s11046-021-00593-7

**Published:** 2021-10-20

**Authors:** Auke W. de Jong, Elaine C. Francisco, João Nóbrega de Almeida, Igor B. Brandão, Felicidade M. Pereira, Pedro H. Presta Dias, Magda M. de Miranda Costa, Regiane T. de Souza Jordão, Duong Vu, Arnaldo L. Colombo, Ferry Hagen

**Affiliations:** 1 Department of Medical Mycology, Westerdijk Fungal Biodiversity Institute, Uppsalalaan 8, NL-3584CT Utrecht, The Netherlands; 2 Department of Medical Microbiology, University Medical Center Utrecht, Utrecht, The Netherlands; 3 Laboratório Especial de Micologia, Disciplina de Infectologia, Universidade Federal de São Paulo, São Paulo, Brazil; 4 Hospital Israelita Albert Einstein, São Paulo, Brazil; 5 Comissão de Controle de Infecção Hospitalar, Hospital de Bahia, Salvador, Brazil; 6 Laboratório Central de Saúde Pública Professor Gonçalo Muniz, Salvador, Brazil; 7 Centro de Informações Estratégicas de Vigilância em Saúde da Bahia, Salvador, Brazil; 8 Agência Nacional de Vigilância Sanitária, Brasília, Brazil; 9 Coordenação Geral de Laboratórios de Saúde Pública, Ministério da Saúde, Brasília, Brazil; 10 Department of Dermatology, Jining No. 1 People’s Hospital, Shandong, China

**Keywords:** *Candida auris*, Emerging pathogen, Nanopore sequencing, De novo genome assembly, Variant calling

## Abstract

*Candida auris* has been reported worldwide, but only in December 2020, the first strain from a COVID-19 patient in Brazil was isolated. Here, we describe the genome sequence of this susceptible *C. auris* strain and performed variant analysis of the genetic relatedness with strains from other geographic localities.

*Candida auris* is an emerging multidrug-resistant pathogen able to cause invasive infection outbreaks in tertiary care hospitals worldwide. *C. auris* has the unique ability to persistently colonize the skin and medical surfaces, making it easily transmissible [1, 2]. Currently, 4 major populations have been described, representing geographic linked clades: South East Asia (clade I), Eastern Asia (clade II), Southern Africa (clade III), South America (clade IV), and a minor fifth lineage (clade V) represented by a single Iranian isolate [2, 3].

*Candida auris* was first reported from Brazil in December 2020 where it was isolated from a COVID-19 patient hospitalized in a reference center at Salvador city. Microsatellite typing showed that the strain (L1537/2020) from the index patient (case one in [4]) is part of the South Asia clade (clade I). Remarkably, the strain was susceptible for all antifungal classes [4]. Whole genome sequencing (WGS) was performed on strain L1537/2020 to enable in-depth analysis of its genetic relatedness with publicly available clade I strains. Additionally, WGS of this strain provides genome data of an antifungal susceptible *C. auris* strain [4, 5].

The strain was cultured in 10 ml peptone glucose broth (LP0040; Oxoid, Basingstoke, UK) and incubated (125 rpm) at 25 °C for 3 days. High-quality genomic DNA was extracted as described before [6]. However, the final step with chloroform/isoamyl-alcohol was replaced by column-based purification using the Fungi/Yeast Genomic DNA kit (catalog nr. 27300; Norgen Biotek, Thorold, ON, Canada) and DNA was eluted in 50 µl molecular grade 1 × IDTE buffer (pH 8.0; IDT, Coralville, IA, USA).

Sequencing was performed on the Oxford Nanopore MinION platform (Oxford Nanopore Technologies, Oxfordshire, UK) using the ligation sequencing kit (SQK-LSK109) and native barcoding kit (EXP-NBD104). The sample was sequenced on the MK1B MinION (MIN-101B) device with a FLO-MIN106 (SpotON R9.4) flowcell using MinKNOWN release 20.10.3.

Raw nanopore reads were basecalled using Guppy (v4.4.1 + 1c81d623j; ONT) with the parameters --flowcell FLO-MIN106 --kit SQK-LSK109 --barcode_kits EXP-NBD104 --device CUDA:0. De novo genome assembly was performed using Flye v2.8.2-b1689 (https://github.com/fenderglass/Flye; [7]) with the parameters --nano-raw < fastq > --out-dir < directory > --genome-size 12.5 m.

The assembly resulted in 15 fragments with a total length of 12,687,478 bp (N_50_ of 2,134,410 bp; largest fragment 4,519,230 bp) with a mean coverage of 300X. However, when the 7 smaller contigs (range 490–4970 bp) were omitted, the mean coverage was 387X. The 8 large contigs (range 27,834–4,519,230 bp; total length 12,675,277 bp) included the circular mitochondrial genome (27,834 bp; 1000X coverage), the remaining nuclear contigs had a coverage of ~ 300X.

Variant calling was performed using a subset of 47 published genomes representing the five *C. auris* clades, including a benchmark set for clade I, (Fig. 1; [3, 8–12]). Sequencing reads were aligned with reference genome B8441 (NCBI accession SRS1558430) using minimap2 for nanopore and bwa v0.7.17-r1188 for Illumina data [13, 14]. Sam-files were sorted and indexed using samtools v1.9 [15]. For nanopore data, longshot was performed on the bam-file to obtain variants of the reference genome [16]. Indels and SNPs with a quality < 20 were removed using vcftools v0.1.15 [17]. For Illumina data, picard (http://broadinstitute.github.io/picard/) was performed to mark duplicates. Variants were then identified with gatk HaplotypeCaller, and SNPs were selected with gatk HaplotypeCaller and filtered with the settings "QD < 2.0||MQ < 40.0||FS > 60.0||SOR > 3.0||MQRankSum < − 12.5 ||ReadPosRankSum < − 8.0" [18]. SNP-files were merged using vcf-merge to produce a fasta-alignment file for all samples with R-package SNPRelate [19]. Different nucleotides within the fasta-alignment file were counted by pairwise SNP-number comparison (Fig. 1, panel A). IQ-TREE v1.6.1 was performed to build a maximum-likelihood tree from the alignment file that was visualized with itol (https://itol.embl.de/) (Fig. 1, panel B; [20]). As a result, 420,164 SNPs were counted. Strains in clade I had ~ 1200–1900 SNPs, while much higher SNP-counts were observed for clade II (~ 45 K), clade III (~ 65 K), clades IV and V (~ 170 K). Pairwise SNP-number comparison showed that all clade I strains were very much similar with only 230–1011 nucleotide differences, except for strain L1537/2020 that had 1893–2089 SNPs compared to the other clade I strains (Fig. 1).

**Fig. 1:**
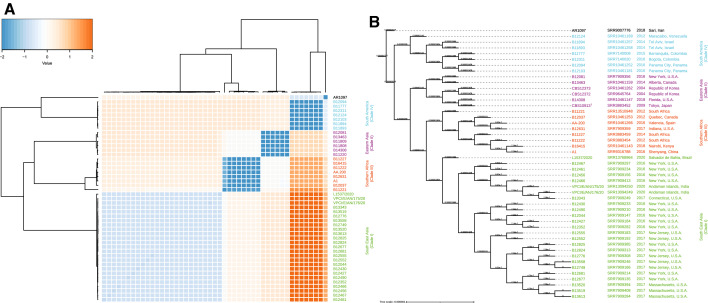
**a** Heatmap visualization of the pairwise SNP-number comparison based on sequences grouped by their z-scores (generated in R package, https://tvpham.github.io/ion/). **b** IQ-TREE generated maximum likelihood phylogenetic analysis using 420,164 SNPs, rooted with strain AR1097 (*C. auris* clade V). Numbers above branches represent branch lengths interpreted as the numbers of nucleotide substitutions per nucleotide site. The heatmap z-scores and the IQ-Tree branch lengths show that clade I strains were very similar to each other, however, strain L1537/2020 had a longer branch length and a high number of SNPs (1893–2089) compared to other strains in clade I

Although strain L1537/2020 belongs to clade I, it is distantly related to all other representatives of that clade, and in contrast it is susceptible to all common antifungals (Fig. 1; [4]). Several mutations are reported to play a role in *C. auris*’ antifungal resistance, viz. *CIS2* (A27T), *ERG3* (W182*, L207I), *ERG11* (Y132F, K143R), *FKS1* (S639P), *MEC3* (A272V), *PEA2* (D367V), *TAC1B* (FS191S, F214S, R495G, S611P), and *UPC2* (M365) [3, 9, 21–23]. None of these mutations are present in the genome of L1537/2020.

Nearly all publicly available *C. auris* genome data was generated by short-read Illumina sequencing [3, 9, 11, 12]. Nonetheless, the relative distant relation of the nanopore-based genome of strain L1537/2020 to other Illumina-sequenced clade I strains cannot be explained by the differences in sequencing technologies. The nanopore flowcell, chemistry and basecalling software used here approaches an accuracy of > 98%. This means that a SNP precision of > 99.9% can be achieved in the case of 50X genome coverage [24]. With the 300X coverage for L1537/2020 it is unlikely that an erroneous mutation was introduced in the assembly. Hence, further studies are needed to investigate the epidemiological and biological impact of the phenotypic and genotypic differences in L1537/2020 versus its multi-drug resistant siblings within clade I.

## Data Availability

https://github.com/vuthuyduong/SNPanalysis.

## Data Availability

Genome data has been deposited in NCBI Genome (BioProject PRJNA704033; BioSample SAMN18028984; Reads SRR13768964).

## Materials Availability

Strain L1537/2020 can be requested via Prof. dr. Arnaldo Lopes Colombo (colomboal@terra.com.br).
